# Comparative Evaluation of Aluminum Oxide and Zinc Oxide Fillers on the Impact Strength and Flexural Strength of Heat-Cure Denture Base Resins: An In Vitro Study

**DOI:** 10.7759/cureus.109653

**Published:** 2026-05-25

**Authors:** Bhavna Bhairi, Girija Dodamani, Priyadarshani Pawar, Dipesh Madge, Rushikesh Wayal, Arun Dodamani, Dimple Mirchandani

**Affiliations:** 1 Department of Prosthodontics, Jawahar Medical Foundation’s Annasaheb Chudaman Patil Memorial Dental College, Dhule, IND; 2 Department of Public Health Dentistry, Jawahar Medical Foundation’s Annasaheb Chudaman Patil Memorial Dental College, Dhule, IND

**Keywords:** aluminum oxide, denture base resins, flexural strength, impact strength, zinc oxide

## Abstract

Background

The long-term performance of heat-cure denture base resins depends largely on their mechanical properties. Heat-cured acrylic resins are widely used in removable prosthodontics because of their practical handling characteristics and acceptable overall clinical utility. The reinforcement of denture base resins with metal oxide fillers has been proposed to improve their resistance to fracture. The present study aimed to evaluate and compare the effects of aluminum oxide and zinc oxide fillers on the impact and flexural strength of heat-cured denture base resins.

Methodology

This in vitro experimental study was conducted at the Department of Prosthodontics, Jawahar Medical Foundation’s Annasaheb Chudaman Patil Memorial Dental College and Hospital, Dhule, Maharashtra, India. The control group (Group 1) (n = 17) consisted of conventional heat-cured denture base resin specimens, Group 2 (n = 17) consisted of aluminum oxide-reinforced specimens, and Group 3 (n = 17) consisted of zinc oxide-reinforced specimens. The flexural strength was evaluated in megapascals (MPa) using a universal testing machine using the three-point bending test method, whereas the impact strength was assessed in kilojoules per square meter (kJ/m²) using the impact test method. Statistical analysis was performed using the Kruskal-Wallis test followed by the Mann-Whitney U test with Bonferroni correction.

Results

The aluminum oxide-reinforced group demonstrated the highest mean flexural strength value (402.94 ± 57.20 MPa), followed by the zinc oxide-reinforced group (164.71 ± 55.24 MPa), whereas the control group showed the lowest value (93.33 ± 22.04 MPa). Similarly, the highest mean impact strength was observed in the aluminum oxide-reinforced group (3.13 ± 1.00 kJ/m²), followed by the zinc oxide-reinforced group (2.22 ± 0.59 kJ/m²) and the control group (1.44 ± 0.31 kJ/m²). Statistically significant differences were observed among all study groups for both flexural and impact strengths (p < 0.001).

Conclusions

The incorporation of aluminum oxide and zinc oxide fillers significantly improved the flexural strength and impact strength of the heat-cured denture base resin compared with the conventional acrylic resin. The aluminum oxide reinforcement demonstrated superior enhancement of the mechanical properties compared to the zinc oxide reinforcement. The reinforcement of denture base resins with metal oxide fillers may improve the durability and fracture resistance of removable prostheses.

## Introduction

Complete dentures are one of the most commonly used treatment modalities for the rehabilitation of completely edentulous patients. The increasing elderly population and prevalence of edentulism have significantly increased the demand for removable prostheses [1]. Among the various denture base materials available, heat-cured acrylic resin continues to be the material of choice because of its favorable esthetics, ease of processing, low cost, satisfactory biocompatibility, and ease of repair [2]. Since its introduction in dentistry, acrylic resin has been extensively used in prosthodontics for the fabrication of denture bases, because it provides acceptable adaptation and functional performance in the oral environment.

Despite their advantages, conventional heat-cured acrylic resins exhibit several mechanical limitations that compromise their long-term clinical success [3]. Denture fractures are among the most common complications encountered in removable prosthodontics and may occur because of accidental dropping, repeated masticatory stresses, flexural fatigue, or poor impact resistance of the denture base material [4]. Studies have shown that a large percentage of dentures fracture within a few years of clinical use, resulting in patient discomfort, increased maintenance, and repeated dental visits [5,6]. Therefore, improving the mechanical properties of denture base resins has become an important area of research in prosthodontics.

Various approaches have been investigated to enhance the strength and durability of denture base materials, including chemical modification of acrylic resin, alteration in polymerization techniques, and reinforcement using fibers, metallic inserts, and ceramic fillers [7-10]. In recent years, nanotechnology has gained considerable attention because the incorporation of nanoparticles into acrylic resins has shown promising improvements in their mechanical and physical properties [7,10]. The reinforcing effect of nanoparticles depends on their size, concentration, distribution, and interaction with the resin matrix.

Among the different reinforcing materials, aluminum oxide has demonstrated favorable properties, such as increased hardness, improved thermal conductivity, and enhanced flexural strength owing to its high strength and stiffness [7]. Zinc oxide nanoparticles have also attracted interest because of their ability to improve mechanical properties along with additional antimicrobial and antifungal benefits [10]. Previous studies evaluating these fillers have reported encouraging results; however, comparative evidence regarding their effect on the impact and flexural strength of denture base resins remains limited [7,10].

Hence, this study aimed to evaluate and compare the effects of aluminum oxide (15 wt%) and zinc oxide (1 wt%) filler incorporation on the flexural strength and impact strength of heat-cured denture base resins, relative to an unreinforced control group, under standardized in vitro laboratory conditions.

## Materials and methods

Study design and setting

This in vitro experimental study was conducted at the Department of Prosthodontics, Jawahar Medical Foundation’s Annasaheb Chudaman Patil Memorial Dental College and Hospital, Dhule, Maharashtra, India, over a period of 12 months. This study was undertaken to evaluate and compare the effects of aluminum oxide and zinc oxide fillers on the impact and flexural strength of heat-cured denture base resins under standardized laboratory conditions. Because the study was performed entirely on fabricated acrylic resin specimens without involving human participants, animal subjects, biological tissues, or patient data, ethical approval was not required for the conduct of the study.

Sample size estimation

**Figure 1:**
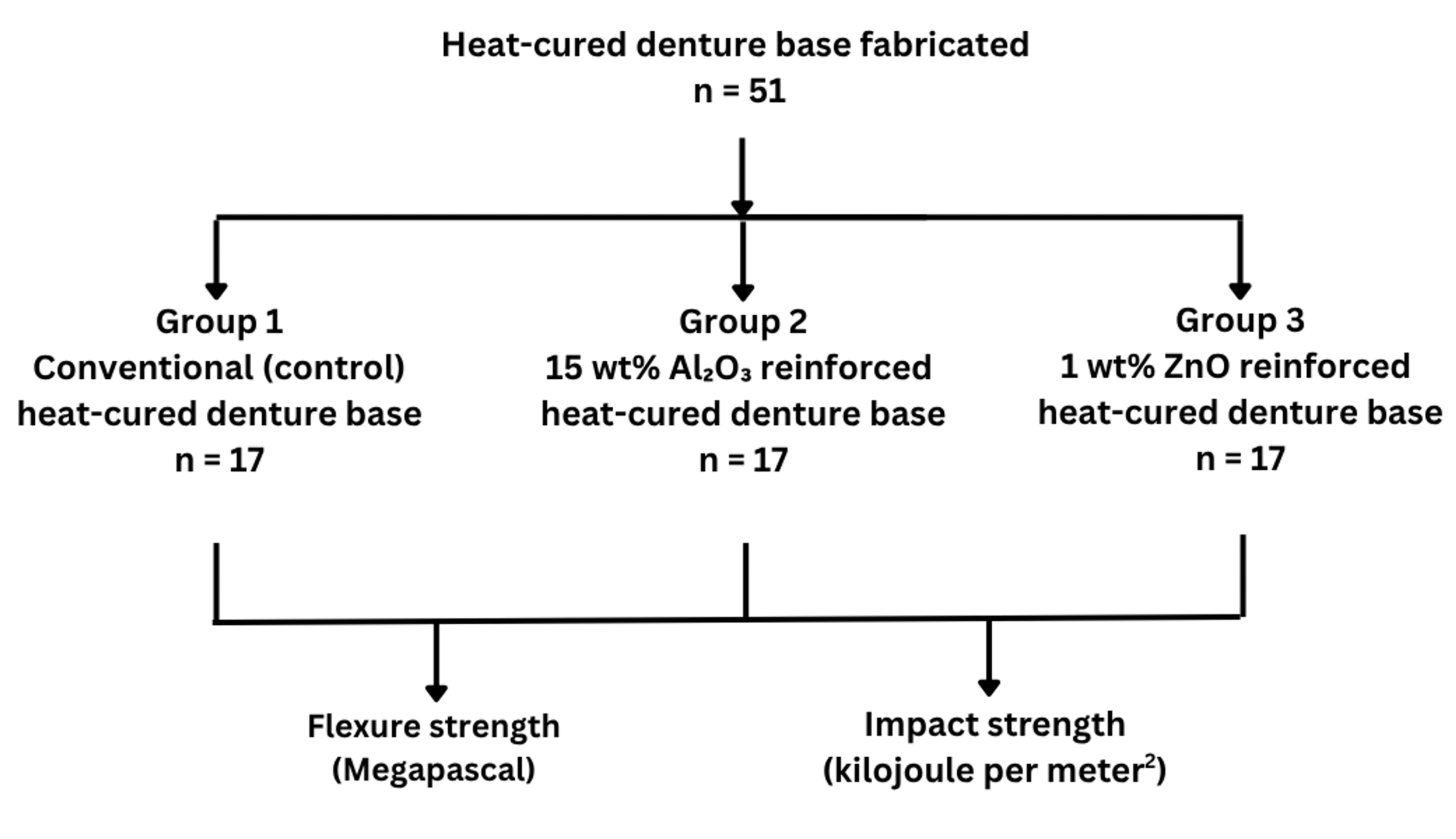
Sample allocation flowchart. The figure was created using Canva (Canva Pty Ltd., Sydney, Australia). ZnO: zinc oxide; Al₂O₃: aluminum oxide

The sample size was estimated using G*Power software version 3.1.9.7 (Heinrich Heine University Düsseldorf, Düsseldorf, Germany). Based on previously published literature evaluating reinforced denture base-resin materials, an effect size of 0.50, a statistical power of 80%, and a confidence interval of 95% were considered [11]. The minimum required sample size was 51. Therefore, 51 specimens were included in this study, with 17 specimens allocated to each study group (Figure 1).

Specimen preparation

A total of 51 customized rectangular acrylic resin specimens measuring 65 × 10 × 2.5 mm were fabricated according to American Dental Association Specification Number 12 for denture base resins. A stainless steel mold with the required dimensions was fabricated for the preparation of the standardized specimens. Heat-cured denture base-resin specimens (Asian Acrylates; Mumbai, Maharashtra, India) were treated as the control group (Group 1). The aluminum oxide and zinc oxide nanoparticles used for reinforcement were procured from the Techinstro Research and Development Center (Chennai, Tamil Nadu, India).

The aluminum oxide (Al₂O₃) nanoparticles used in the present study had an average particle size of 40-60 nm with a purity greater than 99%, whereas the zinc oxide (ZnO) nanoparticles had an average particle size of 20-50 nm with a purity greater than 99%, according to the manufacturer’s specifications (Techinstro Research and Development Center, Chennai, Tamil Nadu, India). Nanoparticles were used in powder form without further surface modification. The concentration of 15 wt% aluminum oxide nanoparticles was selected based on previous studies reporting significant improvement in the flexural strength of PMMA denture base resin at this concentration, whereas 1 wt% ZnO nanoparticles were selected because lower concentrations have demonstrated favorable reinforcement and antimicrobial properties with minimal nanoparticle agglomeration.

Petroleum jelly was applied to a dental flask before the investment procedure. The Type II dental plaster was mixed with water in a rubber bowl according to the manufacturer’s instructions using a mixing spatula and poured into the lower half of the flask. The stainless steel molds were positioned over the unset plaster and allowed to set completely. After setting up the plaster, the separating medium was applied over the surface, and counter-flasking was performed using a dental plaster. Once the plaster had completely set, the flask was opened carefully, and the stainless-steel molds were removed to create mold spaces corresponding to the dimensions of the specimens.

For the control group (n = 17), the acrylic resin was mixed according to the manufacturer’s recommended powder-to-liquid ratio of 3:1 by volume, until the dough stage was achieved. The acrylic resin dough was then packed into the mold spaces with a slight excess of the material. Trial closure was performed using a damp cellophane sheet to remove excess material and to ensure complete adaptation. Following the removal of the excess resin, the final closure of the flask was performed under pressure.

For Group 2 (n = 17), to prepare aluminum oxide-reinforced specimens, aluminum oxide nanoparticles (7.5 g) were incorporated into 50 g of acrylic polymer powder to obtain a concentration of 15% by weight. For Group 3 (n = 17), to prepare the zinc oxide-reinforced specimens, zinc oxide nanoparticles were incorporated into the acrylic polymer powder at a concentration of 1% by weight. The nanoparticles were weighed using a digital analytical balance and initially dry-mixed with the acrylic polymer powder using a sterile glass mortar and pestle for 10 minutes to achieve homogeneous distribution and minimize nanoparticle agglomeration before the addition of monomer liquid. The nanoparticle-polymer powder mixture was visually inspected to ensure uniform dispersion before packing. The packing procedures for the reinforced specimens were performed in a manner similar to those of the control group.

Polymerization and finishing procedures

Following the final closure, all flasks were bench cured at room temperature for 30-60 minutes before polymerization. Polymerization of the acrylic resin specimens was performed in an acrylizing unit using a long curing cycle consisting of heating at 74°C for eight hours, followed by terminal boiling at 100°C for one hour. After polymerization, the flasks were allowed to cool gradually to room temperature before deflation.

The processed acrylic resin specimens were carefully retrieved and examined for surface irregularities, porosities, cracks, and defects. The specimens exhibiting defects were discarded. The acceptable specimens were trimmed using tungsten carbide burs attached to a straight handpiece and laboratory micromotor. The finishing procedures were performed using sequential grades of sandpaper mounted on mandrels to obtain smooth surfaces. The final polishing was performed using pumice and buffing wheels in a polishing unit to obtain uniformly polished specimens suitable for mechanical testing. The dimensions of the finished specimens were verified using a digital caliper.

After finishing and polishing, all specimens were stored in distilled water at 37°C for 48 hours before mechanical testing to simulate oral environmental conditions and permit residual monomer release. All specimens were tested under standardized laboratory conditions at room temperature (23 ± 2°C). Mechanical testing was completed within 48 hours after specimen conditioning.

Evaluation of flexural strength

Flexural strength testing was performed using a universal testing machine (Mecmesin OmniTest 25, PPT Group UK Ltd.) according to American Dental Association Specification Number 12 using the three-point bending test method. Each specimen was positioned horizontally on two supporting rods with a span length of 50 mm. A compressive load was applied centrally at a constant crosshead speed of 5 mm/minute until fracture occurred. The maximum fracture load was recorded in Newtons (N), and flexural strength was calculated in megapascals (MPa) using the following formula: Flexural strength = 3WL/2bd^2^, where W is the maximum fracture load (N), L is the span length (mm), b is the specimen width (mm), and d is the specimen thickness (mm).

Evaluation of impact strength

Impact strength testing was performed using an impact-testing machine (Charpy impact tester, ZwickRoell Pvt. Ltd., Chennai). Each specimen was positioned vertically in the testing apparatus and subjected to a sudden impact load until fracture occurred. The amount of energy absorbed by the specimen during fracture was recorded, and the impact strength was calculated in kilojoules per square meter (kJ/m²).

Statistical analysis

The obtained data were statistically analyzed using SPSS software version 26.0 (IBM Corp., Armonk, NY, USA). Descriptive statistics were expressed as mean and standard deviation, median, and interquartile range for flexural strength and impact strength values. Normality of data distribution was assessed using the Shapiro-Wilk test. As the data demonstrated a non-normal distribution, non-parametric statistical tests were performed. Intergroup comparisons were performed using the Kruskal-Wallis test, followed by pairwise post hoc comparisons using the Mann-Whitney U test with Bonferroni correction. The effect size values were also calculated to determine the magnitude of differences among the study groups. Statistical significance was set at p-values <0.05.

## Results

The normality of data distribution was assessed using the Shapiro-Wilk test. Both flexural strength and impact strength values demonstrated a non-normal distribution across all study groups (p < 0.05); therefore, non-parametric statistical tests were used for intergroup comparisons, as shown in Table 1.

**Table 1:** Shapiro–Wilk test for normality assessment of flexural strength and impact strength among the study groups. The Shapiro-Wilk test was used for normality assessment. P-values <0.05 are considered statistically significant. W = Shapiro-Wilk statistic.

Material group	Flexural strength			Impact strength		
	W statistic	P-value	Distribution	W statistic	P-value	Distribution
Group 1: Acrylic denture base (Control)	0.81	0.001	Non-normal	0.89	0.029	Non-normal
Group 2: Aluminum oxide-reinforced denture base	0.82	0.018	Non-normal	0.80	0.042	Non-normal
Group 3: Zinc oxide-reinforced denture base	0.87	0.028	Non-normal	0.83	0.005	Non-normal

The aluminum oxide-reinforced group demonstrated the highest mean flexural strength value (402.94 ± 57.20 MPa), followed by the zinc oxide-reinforced group (164.71 ± 55.24 MPa), whereas the control group showed the lowest mean flexural strength value (93.33 ± 22.04 MPa). Similarly, the aluminum oxide-reinforced group exhibited the highest mean impact strength (3.13 ± 1.00 kJ/m²), followed by the zinc oxide-reinforced group (2.22 ± 0.59 kJ/m²), whereas the control group demonstrated the lowest mean impact strength (1.44 ± 0.31 kJ/m²), as presented in Table 2.

**Table 2:** Descriptive statistics of flexural strength and impact strength among the study groups. Data presented as mean ± standard deviation (SD) and median with interquartile range (IQR).

Group	N	Flexural strength (MPa)		Impact strength (kJ/m²)	
		Mean ± SD	Median (IQR)	Mean ± SD	Median (IQR)
Group 1: Acrylic denture base (control)	17	93.33 ± 22.04	90.00 (80–100)	1.44 ± 0.31	1.50 (1.20–1.50)
Group 2: Aluminum oxide-reinforced denture base	17	402.94 ± 57.20	400.00 (350–450)	3.13 ± 1.00	3.50 (2.20–4.00)
Group 3: Zinc oxide-reinforced denture base	17	164.71 ± 55.24	150.00 (150–200)	2.22 ± 0.59	2.00 (2.00–3.00)

Intergroup comparisons using the Kruskal-Wallis test revealed statistically significant differences between the three groups for both flexural strength and impact strength (p < 0.001) (Table 3).

**Table 3:** Intergroup comparison of flexural strength and impact strength among the study groups. Kruskal-Wallis test (H) was used for intergroup comparison. P-values <0.05 are considered statistically significant. The effect size represents the magnitude of difference among the groups. *: Statistically significant value.

Statistical test	Test statistic	P-value	Effect size value
Flexural strength	H = 44.464	<0.001*	0.82
Impact strength	H = 33.679	<0.001*	0.61

Pairwise post hoc comparisons using the Mann-Whitney U test with Bonferroni correction demonstrated statistically significant differences between all group combinations for flexural strength and impact strength, as illustrated in Table 4.

**Table 4:** Pairwise comparison of flexural strength and impact strength among the study groups. Mann-Whitney U test with Bonferroni correction used for pairwise comparison. P-values <0.05 are considered statistically significant. U = Mann-Whitney U statistic, adjusted p-value calculated after Bonferroni correction. *: Statistically significant value.

Comparison	Flexural strength		Impact strength	
	U statistic	Adjusted p-value (Bonferroni)	U statistic	Adjusted p-value (Bonferroni)
Control group vs. aluminum oxide-reinforced group	0.00	<0.001*	11.00	<0.001*
Control group vs. zinc oxide-reinforced group	29.00	<0.001*	37.50	<0.001*
Aluminum oxide-reinforced group vs. zinc oxide-reinforced group	288.50	<0.001*	229.00	0.010*

The aluminum oxide-reinforced denture base resin group showed significantly higher flexural strength and impact strength values than both the zinc oxide-reinforced group and the control group. The zinc oxide-reinforced group also demonstrated significantly higher values than those of the control group.

## Discussion

Heat-cured acrylic resin remains the most commonly used denture base material, owing to its acceptable esthetics, ease of manipulation, low cost, and favorable biocompatibility. However, inadequate mechanical properties such as low impact strength and flexural strength continue to be major causes of denture fractures during clinical use [3,4]. Repeated masticatory stresses, accidental dropping of dentures, and flexural fatigue often lead to failure of conventional denture base resins [4]. Therefore, the reinforcement of acrylic resins with filler particles has been extensively investigated to improve their mechanical performance.

The present in vitro study evaluated and compared the effects of aluminum oxide and zinc oxide fillers on the flexural and impact strengths of heat-cured denture base resins. The results of the present study demonstrated statistically significant improvements in both flexural strength and impact strength following the incorporation of aluminum oxide and zinc oxide fillers compared to the conventional heat-cured denture base resin group. Among the reinforced groups, the aluminum oxide-reinforced group exhibited the highest flexural strength and impact strength [12].

The control group exhibited the lowest flexural strength, whereas the aluminum oxide-reinforced group exhibited the highest mean flexural strength. The increased flexural strength observed after the incorporation of aluminum oxide nanoparticles may be attributed to the uniform distribution of filler particles within the acrylic resin matrix, which enhanced stress transfer and reduced crack propagation during loading. Aluminum oxide particles possess high stiffness and hardness, which may improve the resistance of the resin to deformation under functional stresses. In addition, adequate bonding between the filler particles and resin matrix may contribute to improved reinforcement of the denture base material [11]. Dhole et al. [13] evaluated the effect of aluminum oxide filler reinforcement on the flexural strength of different denture base resins and reported a significant improvement in flexural strength following incorporation of 15 wt% aluminum oxide particles.

The findings of the present study are in accordance with those of Ellakwa et al. [11] and Tamore et al. [14], who reported significant improvements in flexural strength following the incorporation of aluminum oxide filler particles into heat-cured acrylic resin. Karci et al. [15] also reported that the addition of aluminum oxide nanoparticles at lower concentrations improved the flexural strength of polymethyl methacrylate denture base resins because of the better dispersion of nanoparticles within the resin matrix. These findings were consistent with those of the present study.

The zinc oxide-reinforced group also demonstrated significantly higher flexural strength than the control group, although the values were lower than those observed in the aluminum oxide-reinforced group. The improvement in flexural strength after the incorporation of ZnO nanoparticles may be related to their nanoscale particle size, which allows better adaptation within the resin matrix and contributes to an improved load distribution during flexural loading. ZnO nanoparticles may also reduce internal defects and void formation within the acrylic resin structure, thereby improving resistance to fracture.

The present findings are comparable with the observations of Vikram and Chander [10], who reported improved flexural strength values following the incorporation of ZnO nanoparticles into denture base resins. Similarly, studies conducted by Asar et al. [16] demonstrated an enhancement in the mechanical properties of heat-cured acrylic resins following reinforcement with metal-oxide fillers. The comparatively lower flexural strength values of the zinc oxide-reinforced resin observed in the present study may be related to differences in the filler concentration, particle interaction, and dispersion characteristics within the polymer matrix.

The impact strength evaluation in the present study also demonstrated significantly higher values in the reinforced group than those in the control group. The aluminum oxide-reinforced group exhibited the highest impact strength values among all the groups. The improvement in impact strength may be attributed to the ability of the filler particles to absorb and dissipate impact energy during sudden loading, thereby preventing crack initiation and propagation within the denture base resin. The incorporation of aluminum oxide particles may also improve the resistance to brittle fracture by increasing the toughness of the acrylic resin matrix [7].

These findings are supported by studies conducted by Asar et al. [16] and Kareem and Moudhaffer [17], who observed improved impact strength in reinforced denture base resins following incorporation of metal oxide nanoparticles. In a systematic review and meta-analysis, Somani et al. [18] also concluded that the reinforcement of polymethyl methacrylate denture base resin significantly improved both flexural strength and impact strength.

The zinc oxide-reinforced group demonstrated a significantly higher impact strength than the control group, although lower values were observed when compared with the aluminum oxide-reinforced group. Zinc oxide nanoparticles possess favorable mechanical characteristics and may improve the energy absorption capacity of acrylic resins. In addition, zinc oxide nanoparticles have been reported to exhibit antimicrobial and antifungal properties, which may provide additional clinical advantages in denture-base applications [19].

The superior mechanical properties demonstrated by the aluminum oxide-reinforced group in the present study suggest that aluminum oxide filler particles may provide more effective reinforcement of heat-cured denture base resins than zinc oxide filler particles [12]. The differences observed between the reinforced groups may be related to the variations in the filler concentration, particle size, filler-matrix interaction, and intrinsic physical properties of the nanoparticles [20].

The filler concentrations selected in the present study were based on previously published literature evaluating reinforcement of denture base acrylic resins [10,12]. Aluminum oxide has previously been investigated at higher concentrations in denture base materials, whereas zinc oxide nanoparticles are commonly used at lower concentrations because higher loading may increase particle agglomeration and adversely affect resin properties. Therefore, although aluminum oxide demonstrated greater improvement in mechanical properties in the present study, the results should be interpreted with caution because the two fillers were evaluated at different concentrations.

The clinical implications of the present study suggest that the reinforcement of heat-cured denture base resins with aluminum oxide and zinc oxide fillers may improve the mechanical durability of removable prostheses and reduce the incidence of denture fractures during clinical use. Improved flexural strength and impact strength may enhance the longevity and serviceability of complete dentures, thereby reducing repair frequency and improving patient satisfaction.

However, the present study has certain limitations. The study was conducted under in vitro conditions, which may not completely reproduce the complex oral environment, including thermal fluctuations, salivary interactions, cyclic masticatory loading, and long-term intraoral aging. Only flexural strength and impact strength were evaluated, whereas other important mechanical and physical properties, such as fatigue resistance, fracture toughness, surface roughness, water sorption, color stability, wear resistance, and biocompatibility, were not assessed. In addition, only one concentration of aluminum oxide and zinc oxide nanoparticles was evaluated. Furthermore, unequal filler concentrations were used between the experimental groups (15 wt% aluminum oxide and 1 wt% zinc oxide), which may limit direct comparability between the reinforcing materials and should be considered while interpreting the comparative outcomes. Nanoparticle characterization methods such as scanning electron microscopy, particle size analysis, or assessment of filler dispersion within the resin matrix were not performed, which may influence the interpretation of the reinforcement mechanism.

Although the Charpy impact test is a standardized laboratory method for evaluating impact resistance, it may not completely simulate the multidirectional impact forces encountered clinically during accidental denture dropping. Additionally, while statistically significant differences were observed among the study groups, the clinical significance of these improvements relative to established ISO standards and clinically acceptable thresholds for denture base resins was not evaluated. Future studies should therefore include standardized nanoparticle characterization, equalized filler concentrations, long-term thermocycling and fatigue testing, finite element analysis, and in vivo clinical assessment to better determine the practical applicability of nanoparticle-reinforced denture base resins.

Future studies should evaluate the long-term clinical performance of reinforced denture base resins under simulated oral conditions, including cyclic fatigue loading, thermal cycling, water sorption, color stability, and biocompatibility assessment. Further investigations comparing different nanoparticle concentrations, particle sizes, and surface treatments may help optimize the reinforcing potential of metal oxide fillers. In vivo clinical trials are also required to determine patient-centered outcomes and long-term durability of reinforced prostheses.

## Conclusions

Within the limitations of the present in vitro study, the incorporation of aluminum oxide and zinc oxide fillers significantly improved the flexural strength and impact strength of heat-cured denture base resins compared with conventional acrylic resin. The aluminum oxide-reinforced group demonstrated higher mechanical strength values than the zinc oxide-reinforced group under the concentrations evaluated in the present study. However, direct comparison between the reinforcing efficacy of the two fillers should be interpreted with caution because unequal filler concentrations were used between the experimental groups (15 wt% aluminum oxide and 1 wt% zinc oxide). Therefore, the present study cannot conclusively establish the superiority of one filler over the other. The findings of this study suggest that nanoparticle reinforcement may enhance the mechanical performance of denture base resins under laboratory conditions. However, the present investigation was limited to in vitro mechanical testing and did not evaluate clinical performance, long-term intraoral durability, patient-centered outcomes, or functional longevity of dentures. Further studies using standardized filler concentrations, thermocycling protocols, fatigue analysis, and long-term clinical evaluation are necessary before definitive clinical recommendations can be made.
